# Variability in donor leukocyte counts confound the use of common RNA sequencing data normalization strategies in transcriptomic biomarker studies performed with whole blood

**DOI:** 10.1038/s41598-023-41443-4

**Published:** 2023-09-19

**Authors:** Grant C. O’Connell

**Affiliations:** 1 Molecular Biomarker Core, Case Western Reserve University, 10900 Euclid Avenue, Cleveland, OH 44106-4904 USA; 2 School of Nursing, Case Western Reserve University, Cleveland, OH USA

**Keywords:** Gene expression profiling, RNA sequencing, RNA sequencing

## Abstract

Gene expression data generated from whole blood via next generation sequencing is frequently used in studies aimed at identifying mRNA-based biomarker panels with utility for diagnosis or monitoring of human disease. These investigations often employ data normalization techniques more typically used for analysis of data originating from solid tissues, which largely operate under the general assumption that specimens have similar transcriptome composition. However, this assumption may be violated when working with data generated from whole blood, which is more cellularly dynamic, leading to potential confounds. In this study, we used next generation sequencing in combination with flow cytometry to assess the influence of donor leukocyte counts on the transcriptional composition of whole blood specimens sampled from a cohort of 138 human subjects, and then subsequently examined the effect of four frequently used data normalization approaches on our ability to detect inter-specimen biological variance, using the flow cytometry data to benchmark each specimens true cellular and molecular identity. Whole blood samples originating from donors with differing leukocyte counts exhibited dramatic differences in both genome-wide distributions of transcript abundance and gene-level expression patterns. Consequently, three of the normalization strategies we tested, including median ratio (MRN), trimmed mean of m-values (TMM), and quantile normalization, noticeably masked the true biological structure of the data and impaired our ability to detect true interspecimen differences in mRNA levels. The only strategy that improved our ability to detect true biological variance was simple scaling of read counts by sequencing depth, which unlike the aforementioned approaches, makes no assumptions regarding transcriptome composition.

## Introduction

The advent of whole transcriptome profiling techniques, along with the push towards personalized medical treatments, has led to the clinical emergence of multi-analyte algorithmic molecular diagnostics which target mRNA. The peripheral whole blood is an attractive source of candidate biomarkers to develop such tests, as it is relatively easy to sample, and exhibits altered gene expression in several disease states via the peripheral immune response^1^. Currently, diagnostics targeting whole blood gene expression signatures are being assessed for a variety of clinical uses including risk stratification in cancer^2^, early detection of chronic infectious disease^3^, diagnosis of various neurologic disorders^4, 5^, and organ transplant monitoring^6, 7^. Given the potential, countless biomarker discovery investigations are now performed involving high-throughput gene expression profiling of whole blood, and even more are carried out via secondary analyses and meta-analyses of the resultant data.

Because the translational goal of these studies is typically development of biomarker panels that could ultimately be measured in the blood with minimal specimen handling and post-analytical data processing in a clinical laboratory setting, often using lower throughput techniques such as qRT-PCR, the focus is largely on identifying diagnostically targetable absolute differences in mRNA levels that are directly observable at the bulk tissue level. This differs in some regards from the studies which use whole blood transcriptomics to investigate physiologic or pathophysiologic mechanisms, in that the latter may be more focused on making inferences about the underlying nuclear transcriptional state of the constitute blood cells as opposed to best quantifying the true RNA levels in a sample. Given this fundamental difference, it is important that the methodology employed in biomarker-centric investigations is specifically tailored to the application.

A large majority of the data collected or used in transcriptomic biomarker discovery studies of whole blood today is now generated via next generation RNA sequencing (RNA-seq). One of the fundamental steps in the analysis of bulk RNA-seq data generated from any tissue is normalization of the raw read counts^8^. While the primary goal of normalization is remove technical variance, if performed incorrectly, normalization can introduce unintended artifacts and impair the ability to detect true differences in RNA levels^9^. For example, prior studies have demonstrated that the use of different normalization approaches can generate highly discordant results from the same dataset^10, 11^, and that in some circumstances, normalized data can perform worse for disease diagnosis than the raw data itself^12^. Thus, selection of an appropriate data normalization strategy is critical to increase the odds of identifying gene expression signatures with true diagnostic utility in RNA-seq based biomarker discovery workflows.

Despite the importance of normalization and potential impact on results, many biomarker discovery investigations performed using RNA-seq data generated from whole blood simply employ the default normalization strategies deployed in the most commonly used RNA-seq data analysis tools such as the DESeq2^13^ and EdgeR^14^ packages for R^15^. However, many of the assumptions underlying these commonly used normalization strategies may not hold true in whole blood, especially if the goal of analysis is to identify purely diagnostic differences in mRNA levels between disease states. For example, a majority of widely used global scaling normalization techniques, including median-ratio normalization (MRN)^13^ and trimmed mean of m-values (TMM) normalization^14^, work under the assumption that a majority of genes are not differentially expressed between specimens^16^. Furthermore, other commonly used normalization strategies, including quantile normalization, assume that all specimens have similar global distributions of transcript levels^16^. Both of these key assumptions are likely to be violated when working with data generated from whole blood if there are inter-donor differences in leukocyte counts.

Unlike solid tissues which are relatively static in terms of cellular composition, the cellular composition of whole blood is highly dynamic and can differ substantially between donors. With the exception of hemoglobin transcripts, which are often selectively depleted prior to gene expression profiling^17^, virtually all of the of the mRNA in whole blood originates from white blood cells; furthermore, a majority of this mRNA is contributed by neutrophils and lymphocytes specifically, as they collectively make up well over 90% of the circulating leukocyte pool^18^. However, circulating proportions of neutrophils and lymphocytes can differ significantly between donors. For example, the ratio of circulating neutrophils to circulating lymphocytes, commonly referred to as the neutrophil-to-lymphocyte ratio (NLR), ranges from roughly 4:1 (4.0) to 1:2 (0.5) in healthy adults^19^, with more extreme values observed in a wide variety of disease states^20–26^.

Because the predominate leukocyte subpopulations found in circulation each carry highly unique transcriptomes^27^, any shift in white blood cell differential has the potential to result in dramatic changes in gene expression at the level of whole blood, even in the absence of true transcriptional differences in gene expression at the level of individual cells^28^; given their outsized contributions to the total pool of whole blood RNA, it is likely that even the normal inter-individual heterogeneity in neutrophil-to-lymphocyte ratio, much less that which is observed in disease states, results in near genome-wide differences in whole blood transcript levels that violate the assumptions of the aforementioned normalization approaches. Thus, the use of these normalization approaches when analyzing whole blood data in mRNA-based biomarker discovery studies has the potential to generate artifactual false positives or obscure true biologic differences in transcript levels that have diagnostic potential.

Despite this potential confound, no studies have formally investigated the effects of inter-donor differences in white blood cell counts on the composition of the whole blood transcriptome within the context of implications for data normalization in RNA-seq based biomarker discovery workflows. In this study, we used RNA sequencing to generate genome-wide gene expression profiles of whole blood specimens collected from a cohort of human subjects recruited in an emergency medicine setting, and used white blood cell differential data collected from the same blood draw with flow cytometry to examine the influence of shifts in neutrophil-to-lymphocyte ratio on global transcriptome composition. Then, we examined the effect of four commonly used data normalization approaches on our ability to detect true inter-specimen biological variance, using the white blood cell differential data to benchmark each specimens true molecular and cellular identity.

## Results

### Isolated neutrophils and lymphocytes exhibit dramatic differences in transcriptome composition

In order to better understand the potential effects of shifts in neutrophil-to-lymphocyte ratio on the composition of the whole blood transcriptome, we first compared the transcriptomes of the respective cell populations themselves using publically available gene expression data. In particular, we assessed differences in overall distributions of transcript abundance, as well as gene-level differential expression, using genome-wide data generated from isolated human neutrophils and lymphocytes via both next generation sequencing and microarray. Data generated via both technologies was used in order to give the clearest picture of transcriptome differences independent of platform-specific caveats such as differences in dynamic range and composition biases.

**Figure 1:**
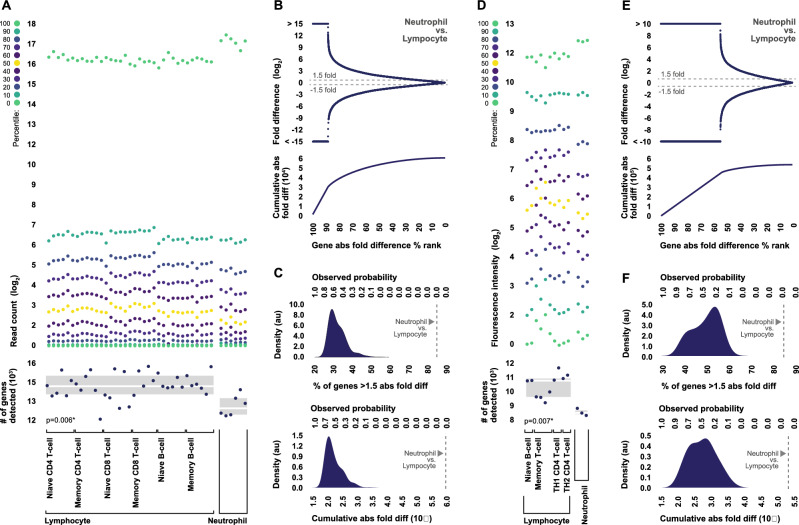
Comparison of transcriptome composition between isolated neutrophils and lymphocytes. (**A**) Genome-wide distributions of transcript abundance, as well as the total number of genes with detectable transcript, in samples of isolated human neutrophils (n = 6) and lymphocytes (n = 35) profiled by RNA-sequencing. Transcript abundance was quantified using TPM values. The total number of genes detected was statistically compared between cell types using Mann Whitney U-Test. Boxplots indicate median and interquartile range. (**B**) Fold differences in expression levels between neutrophil and lymphocyte samples profiled with RNA sequencing for all reliably detected genes, ordered from highest to lowest absolute fold difference, along with the cumulative absolute fold difference. Fold differences were calculated from median TPM values. (**C**) Probability density distributions generated from the percentages of genes exhibiting at least 1.5-fold difference, as well the genome-wide cumulative absolute fold differences, observed in 1000 comparisons of pseudo-randomly selected groups of n = 6 and n = 35 sequencing samples constrained to contain equivalent proportions of neutrophil and lymphocyte samples, compared to the values observed when comparing neutrophil and lymphocyte samples specifically. (**D**) Genome-wide distributions of transcript abundance, as well the total number of genes with detectable transcript, in samples of isolated human neutrophils (n = 3) and lymphocytes (n = 10) profiled by microarray. Transcript abundance was quantified using raw background corrected fluorescence intensities. The total number of genes detected was statistically compared between cell types using Mann Whitney U-Test. Boxplots indicate median and interquartile range. (**E**) Fold differences in expression levels between neutrophil and lymphocyte samples profiled with microarray for all reliably detected genes, ordered from highest to lowest absolute fold difference, along with the cumulative absolute fold difference. Fold differences were calculated from median intensity values. (**F**) Probability density distributions generated from the percentages of genes exhibiting at least 1.5-fold difference, as well the genome-wide cumulative absolute fold differences, observed in 200 comparisons of pseudo-randomly selected groups of n = 3 and n = 10 microarray samples constrained to contain equivalent proportions of neutrophil and lymphocyte samples, compared to the values observed when comparing neutrophil and lymphocyte samples specifically.

Expectedly, both sequencing data and microarray data revealed dramatic differences in transcriptome composition between cell types. Across both platforms, distributions of mRNA abundance in neutrophil samples were dominated by a small number of highly expressed transcripts, characterized by higher upper percentiles, lower medians, and a smaller number of contributing genes, whereas distributions of mRNA abundance in lymphocyte samples were more normal and more diverse, characterized by lower upper percentiles, higher medians, and a larger number of contributing genes (Fig. 1A,D).

In terms of gene-level differential expression, we observed near genome-wide differences, as 84.1% and 84.4% of reliably detected genes exhibited at least a 1.5-fold difference in expression levels between cell types in the sequencing and microarray datasets respectively (Fig. 1B,E). In order to contextually evaluate the collective magnitude of these observed differences, a set of permutation analyses were performed using both the sequencing and microarray datasets in which 1000 and 200 comparisons each were generated between pseudo-randomly selected groups of samples constrained to contain equivalent proportions of neutrophil and lymphocyte samples. The median number of genes exhibiting at least a 1.5-fold difference in expression levels across these permutation comparisons were 34.5% and 48.2% in the sequencing and microarray datasets respectively, and in both instances, not a single permutation comparison produced a greater number of differentially expressed genes than we observed in the our actual comparisons of neutrophil and lymphocyte samples (Fig. 1C,F).

### Shifts in neutrophil-to-lymphocyte ratio globally alter the transcriptome composition of whole blood

Given the substantial differences we observed between the transcriptomes of isolated neutrophils and lymphocytes, we would expect that differences in donor neutrophil-to-lymphocyte ratio would result in corresponding differences in the transcriptional composition of whole blood. To explore this possibility, we used RNA sequencing to generate genome-wide gene expression profiles from human whole blood specimens sampled from a cohort of 138 patients presenting to the emergency department of an academic hospital with a variety of medical conditions (Table 1). We then examined the relationship between transcriptome composition and donor neutrophil-to-lymphocyte ratio, which was calculated from white blood cell differential data generated with flow cytometry from parallelly drawn specimens. Specifically, we assessed the correlation between donor neutrophil-to-lymphocyte ratio and the observed genome-wide distribution of transcript abundance, as well as gene-level differential expression between specimens originating from donors with neutrophil-to-lymphocyte ratios in the upper and lower quartiles.

**Table 1:** Donor clinical and demographic characteristics.

	n = 138
**Demographic characteristics:**	
Age *median* (range)	64 (20–96)
Female *n* (%)	66 (47.8)
Male *n* (%)	72 (52.2)
White *n* (%)	121 (87.7)
African American *n* (%)	15 (10.9)
Asian *n* (%)	1 (0.7)
American Indian *n* (%)	1 (0.7)
Hispanic *n* (%)	41 (29.7)
**Leukocyte counts:**	
% Neutrophil count *median* (range)	63.8 (20.8–91.1)
% Lymphocyte count *median* (range)	24.3 (3.8–57.4)
% Monocyte count *median* (range)	7.2 (2.5–30.2)
% Eosinophil count *median* (range)	1.5 (0.1–7)
Neutrophil-to-lymphocyte ratio *median* (range)	2.57 (0.48–23.98)

**Figure 2:**
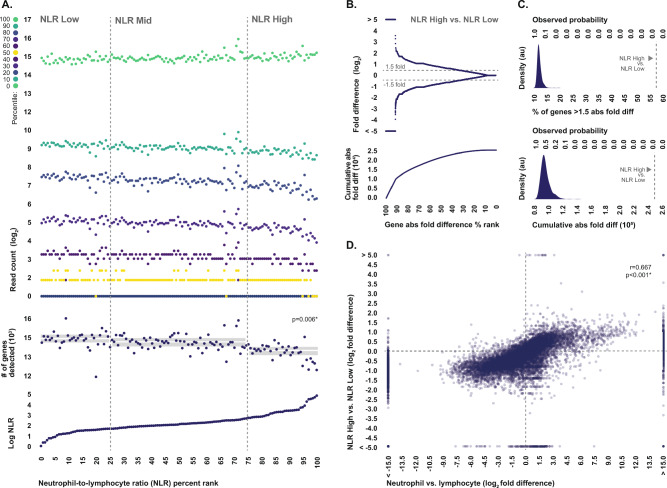
Comparison of whole blood transcriptome composition between donors with differing neutrophil-to-lymphocyte ratios. (**A**) Genome-wide distributions of raw read counts generated from whole blood specimens, as well as the total number of genes with detectable transcript, ordered by donor neutrophil-to-lymphocyte ratio. The number of detected genes was statistically compared between specimens from donors with neutrophil-to-lymphocyte ratios in the lower quartile (n = 35), middle two quartiles (n = 68) , and upper quartile (n = 35) with Kruskal–Wallis rank test. Boxplots indicate median and interquartile range. (**B**) Fold differences in raw read counts between specimens from donors with neutrophil-to-lymphocyte ratios in the upper and lower quartiles for all reliably detected genes, ordered from highest to lowest absolute fold difference, along with the cumulative absolute fold difference. Fold differences were calculated from median values. (**C**) Probability density distributions generated from the percentages of genes exhibiting at least 1.5-fold difference, as well the genome-wide cumulative absolute fold differences, observed in 1000 comparisons of pseudo-randomly selected groups of n = 35 and n = 35 specimens constrained to have a less than a 5% difference in donor neutrophil-to-lymphocyte ratio, compared to the values observed when comparing specimens from donors with neutrophil-to-lymphocyte ratios in the upper and lower quartiles. (**D**) Relationship between gene-level fold differences observed in whole blood between donors with neutrophil-to-lymphocyte ratios in the upper and lower quartiles, and those observed between isolated neutrophils and lymphocytes. Correlation strength and statistical significance was assessed via Spearman’s rho. *Statistically significant.

Distributions of unnormalized read counts associated with whole blood specimens from donors with low neutrophil-to-lymphocyte ratios looked highly similar to the transcript distributions we observed in isolated lymphocytes, whereas distributions of unnormalized read counts associated with whole blood specimens from donors with high neutrophil-to-lymphocyte ratios looked highly similar to the transcript distributions we observed in isolated neutrophils (Fig. 2A).

Furthermore, in terms of gene-level differential expression, 58.3% of reliably detected genes exhibited at least a 1.5-fold difference in unnormalized read counts between whole blood specimens originating from donors with neutrophil-to-lymphocyte ratios in the upper and lower quartiles (Fig. 2B). In order to establish whether these fold differences were attributed to true biological differences as opposed to technical artifacts, we compared them to the fold differences we observed in our prior analysis of isolated neutrophils and lymphocytes. Fold differences were highly-correlated genome-wide (r = 0.677, p < 0.001, Fig. 2D), suggesting that the widespread gene-level differences in read counts we observed were attributed to true differences in RNA levels resulting from the underlying differences in neutrophil and lymphocyte composition. In order to contextually evaluate the collective magnitude of these differences, we performed a permutation analysis in which 1000 comparisons were generated between pseudo-randomly selected groups of specimens constrained to have similar composition in terms of donor neutrophil-to-lymphocyte ratio. The median percentage of genes exhibiting at least a 1.5-fold difference in read counts across these permutation comparisons was 11.5%, and not a single permutation comparison produced a greater number of differential read counts than we observed in our actual comparison of specimens originating from donors with high and low neutrophil-to-lymphocyte ratios (Fig. 2C).

The results of these analyses demonstrate the profound effect that shifts in donor neutrophil-to-lymphocyte ratio have on composition of the whole blood transcriptome, and in fact, suggest that neutrophil-to-lymphocyte ratio is likely by a wide margin the single largest determinant of the overall pattern of gene expression observed at the level of whole blood.

### Commonly employed normalization approaches mask true biologic variance when applied to data generated from whole blood specimens with differing cellular composition

The dramatic shifts in the composition of the whole blood transcriptome we observed as a result of shifts in neutrophil-to-lymphocyte ratio suggest that the underlying assumptions central to many of the most common techniques used for normalization of RNA sequencing data, especially assumptions related to genome-wide distributions of transcript abundance and gene-level differential expression trends, would be violated in any analysis involving data originating from specimens with differing neutrophil and lymphocyte composition. Thus, it is likely that the use of these normalization techniques in such situations could interfere with the ability to detect true biological variance. In order to explore this possibility, we performed a series of analyses to examine the impact of four commonly used data normalization approaches on our ability to detect true inter-specimen biological variance, using the white blood cell differential data to benchmark each specimens true molecular and cellular composition.

**Figure 3:**
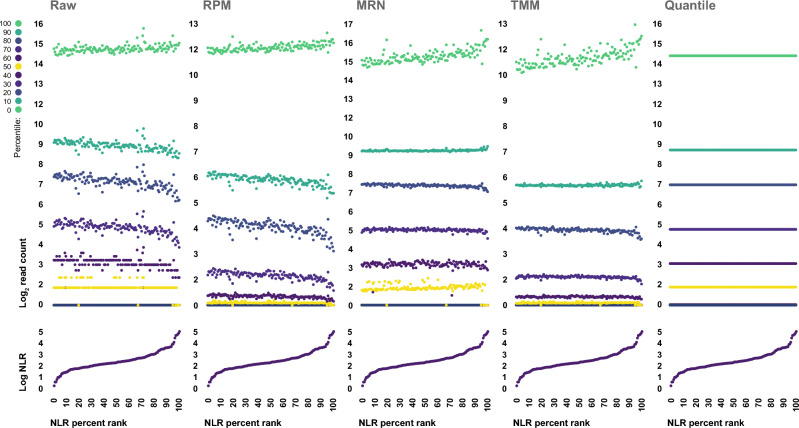
Effects of normalization on whole blood read count distributions. Genome-wide distributions of read counts generated from whole blood specimens (n = 138) both before (Raw) and after scaling by sequencing depth (RPM), median ratio normalization (MRN), trimmed mean of M-values (TMM) normalization, and quantile normalization, ordered by donor neutrophil-to-lymphocyte ratio.

The following four data normalization approaches were evaluated: simple scaling of read counts by sequencing depth to yield normalized counts in terms of reads per million mapped (RPM), median-ratio normalization (MRN), trimmed mean of m-values (TMM) normalization, and quantile normalization. The resultant pre and post-normalization read count distributions are depicted in Fig. 3. With the exception of simple scaling by read depth, each normalization strategy that was tested disrupted the clear relationship between read count distribution and donor neutrophil-to-lymphocyte ratio that we had observed in the raw data, suggesting that their use removed or obscured a significant amount of the true biological structure of the data.

**Figure 4:**
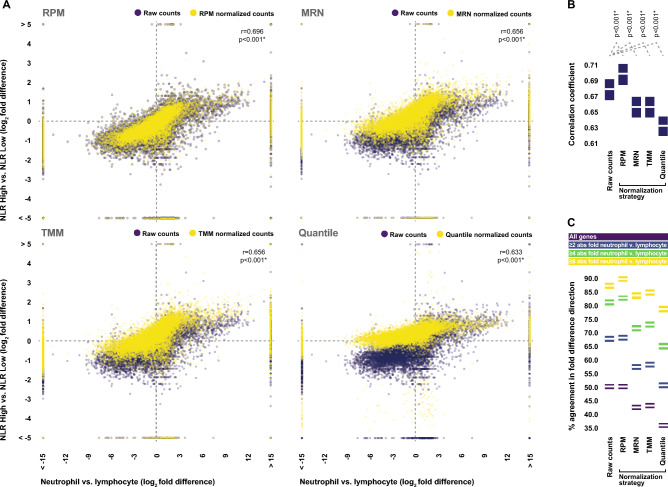
Effects of normalization on the ability to detect variance in whole blood gene expression levels associated with underlying variance in sample cellular composition. (**A**) Relationship between gene-level fold differences in whole blood transcript levels observed between donors with neutrophil-to-lymphocyte ratios in the upper and lower quartiles, and those observed between isolated neutrophils and lymphocytes, both before (Raw) and after adjusting the whole blood read counts by either simple sequencing depth correction (RPM), median ratio normalization (MRN), trimmed mean of M-values (TMM) normalization, and quantile normalization. The strength and statistical significance of correlations was assessed via Spearman’s rho. (**B**) Statistical comparisons of the correlation coefficient observed with raw whole blood read counts and those observed following adjustment via each normalization strategy. Boxplots indicate 95% confidence intervals. Confidence intervals and p-values were generated via the percentile bootstrap method using 1000 bootstrap samples. (**C**) Percent agreement in the direction of gene-level fold differences in whole blood transcript levels observed between donors with neutrophil-to-lymphocyte ratios in the upper and lower quartiles, and those observed between isolated neutrophils and lymphocytes, both before and after adjusting whole blood read counts via each normalization strategy. Percent agreement is presented for all genes, as well as subsets of genes which respectively exhibited at least a twofold, fourfold, or eightfold difference in expression levels between isolated neutrophils and lymphocytes. Boxplots indicate 95% confidence intervals. Confidence intervals were generated via the percentile bootstrap method using 1000 bootstrap samples. *Statistically significant.

In order to examine this potential effect further, we assessed the impact of the tested normalization strategies on our ability to detect expected inter-specimen differences in gene-level mRNA expression. Given that our collective prior analyses demonstrated that whole blood mRNA levels are largely dependent on the cellular composition of the specimen, and that specimens with differing cellular composition exhibit inter-specimen gene-level differential expression patterns which are highly reflective of those that exist between their isolated constitute cell populations, we assessed the impact of each normalization strategy on the correlation that we previously observed between the genome-wide fold differences in raw read counts yielded from comparison of whole blood specimens from donors with high and low neutrophil-to-lymphocyte ratios, and those yielded from comparison of isolated neutrophils and lymphocytes. Purely in terms of correlation coefficient, simple scaling of the raw read counts by sequencing depth significantly improved the strength of the observed correlation, however, the use of every other normalization strategy weakened it (Fig. 4A,B). Furthermore, simple scaling of the raw read counts by sequencing depth resulted in improved directional agreement in fold difference values, whereas the use of every other normalization strategy introduced discordance. These effects were most pronounced when looking at genes which exhibited higher fold differences in expression levels between isolated neutrophils and lymphocytes, and thus whose whole blood transcript levels should be most affected by shifts in the neutrophil-to-lymphocyte ratio (Fig. 4C).

**Figure 5:**
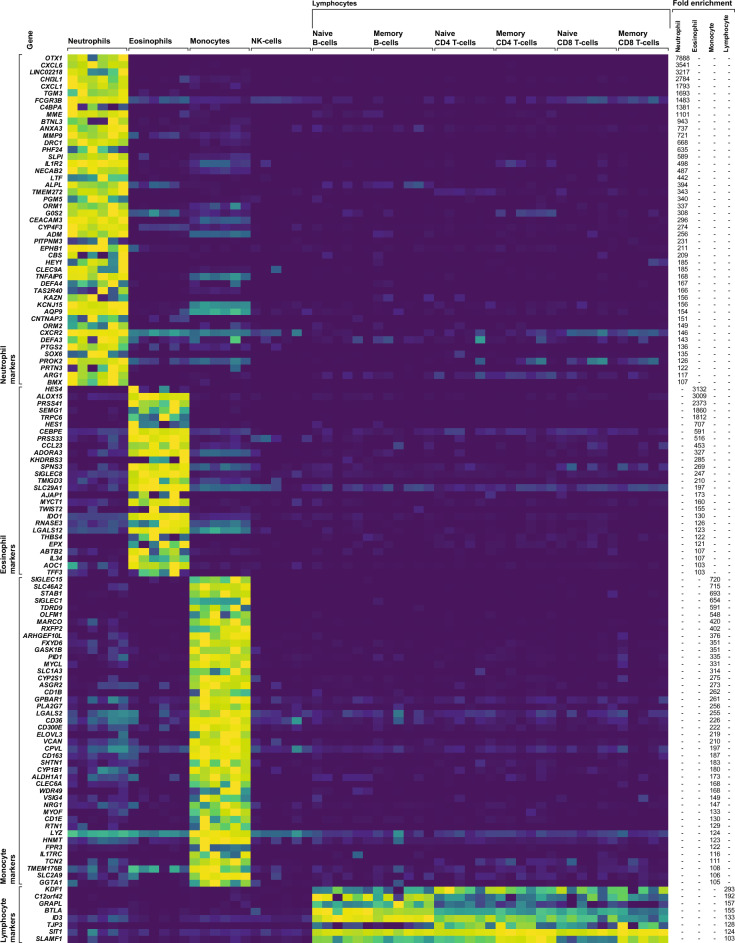
WBC4.126 marker gene list used for cellular deconvolution. Expression levels, of the 126 genes which make up the WBC4.126 marker gene list in isolated neutrophils (n = 6), eosinophils (n = 6), monocytes (n = 6), natural killer cells (n = 6), and lymphocytes (n = 35). The degree of fold enrichment in the cell type each gene is designated to represent is indicated relative to all other cell types. Indicated expression levels represent TPM values, and fold enrichment was calculated from leukocyte subpopulation weighted medians.

**Figure 6:**
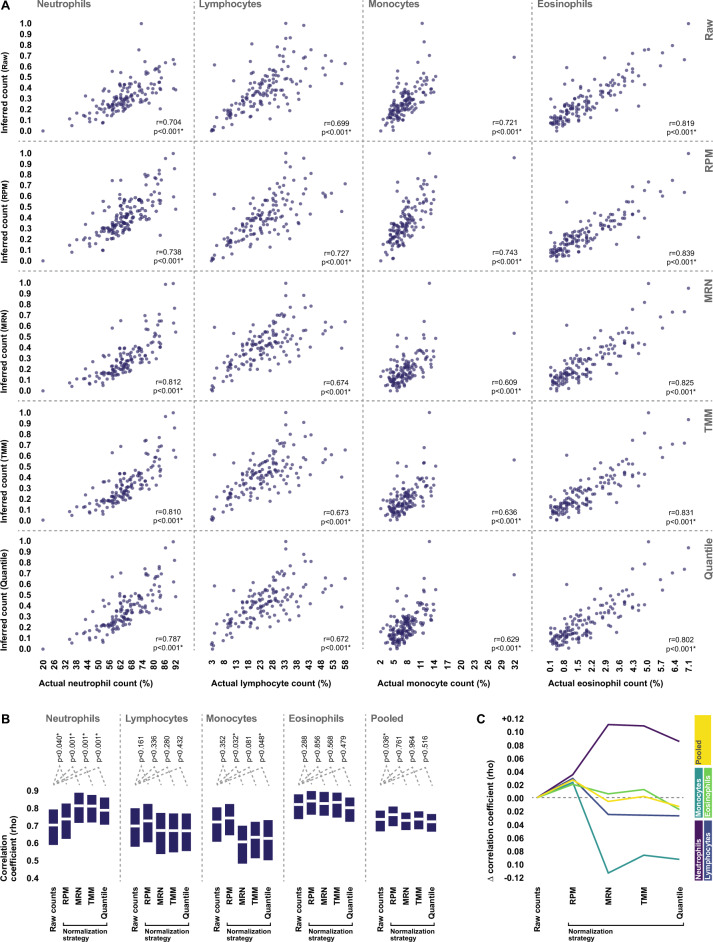
Effects of normalization on the ability to infer sample composition from whole blood gene expression data using cellular deconvolution. (**A**) Correlations between actual donor peripheral blood leukocyte counts measured by flow cytometry, and those inferred from whole blood gene expression data both before (Raw) and after adjusting read counts by either simple sequencing depth correction (RPM), median ratio normalization (MRN), trimmed mean of M-values (TMM) normalization, and quantile normalization. The strength and statistical significance of correlations was assessed via Spearman’s rho. (**B**) Statistical comparisons of the correlation coefficient observed with inferred cell counts generated from raw whole blood read counts, and those generated following each normalization strategy, for each cell type. Pooled correlation coefficients represent the average correlation coefficient observed across all cell types. 95% confidence intervals and p-values were generated via the percentile bootstrap method using 1000 bootstrap samples. (**C**) The change in correlation coefficient associated with each normalization strategy for each cell type, relative to the correlation coefficient observed when performing deconvolution directly from raw read counts. *Statistically significant.

Finally, we assessed the impact of each normalization strategy on our ability to bioinformatically infer the true cellular composition of each whole blood specimen using gene expression data. To do this, we leveraged the same publically available RNA-sequencing dataset of isolated human leukocyte subpopulations that we employed in our prior analyses to identify a list of 126 marker genes (hereafter referred to as WBC4.126, Supplementary File 1) whose expression levels are highly enriched in either neutrophils, lymphocytes, monocytes, or eosinophils (Fig. 5). We then applied a principal components analysis-based deconvolution approach to generate inferred counts for the aforementioned cell types using both the raw and normalized whole blood expression levels of these marker genes. The resultant inferred cell counts were then compared to the actual donor white blood cell differentials generated via flow cytometry, and the effect of each normalization approach on the accuracy of deconvolution was assessed. Inferred cell counts generated from the raw data were highly correlated with actual donor cell counts. Simple scaling of the raw read counts by sequencing depth resulted in an improvement in deconvolution accuracy in the case of every leukocyte subpopulation, whereas normalization via all other strategies resulted in a decreased deconvolution accuracy in the case of either all or nearly every leukocyte subpopulation (Fig. 6A,C).

The results of these analyses (summarized in Table 2) suggest that with the exception of simple correction for sequencing depth, all of the data normalization strategies that we evaluated significantly interfere with the ability to detect true biological variance in the presence of inter-specimen differences in cellular composition.

**Table 2:** Summary of observed performance of tested normalization strategies.

	Expected global distributions of transcript abundance maintained following normalization:	Effect of normalization on ability to detect expected gene-level fold differences:	Effect of normalization on ability to infer sample cellular composition:
RPM normalization:	Yes	Improved	Improved
MRN normalization:	No	Reduced	Reduced
TMM normalization:	No	Reduced	Reduced
Quantile normalization:	No	Reduced	Reduced

## Discussion

In this study, our goal was to examine how shifts in donor leukocyte counts alter the transcriptional composition of whole blood, and determine how these alterations influence the performance of commonly employed RNA-sequencing data normalization strategies in terms of detecting true biological variance in mRNA levels. Our results demonstrate that shifts in donor leukocyte counts profoundly alter the composition of the whole blood transcriptome, and that many of the data normalization strategies that are currently being deployed in sequencing-based transcriptomic biomarker discovery studies of whole blood are likely to significantly interfere with the ability to detect true biological variance in transcript levels in the presence of inter-donor differences in leukocyte counts as a result of said alterations.

A majority of the most commonly-employed RNA sequencing data normalization approaches used today operate under at least one of the following two assumptions: that either genome-wide distributions of transcript levels are similar between specimens, or a majority of genes are not differentially expressed between specimens^16^. Our results demonstrate that not only can whole blood specimens with differing cellular composition have dramatically different global distributions of transcript levels, but that they can also exhibit near genome-wide gene-level differential expression, phenomena which would clearly result in violation of the aforementioned assumptions in any analysis involving specimens originating from donors with variable leukocyte counts. Our findings further suggest that the use of these normalization methods in the presence of these violations can introduce significant analytical confounds. Of the four normalization approaches we evaluated in our benchmarking analysis, three, including MRN, TMM, and quantile normalization, noticeably masked the true biological structure of the data, and impaired our ability to detect true interspecimen differences in whole blood transcript levels. Thus, the use of these normalization approaches or others which are based on similar assumptions is not recommended in future biomarker discovery investigations performed with data generated from whole blood, given the significant heterogeneity in leukocyte counts that is present across even healthy populations^19^, much less populations with disease^20–26^. The only normalization strategy that we evaluated that improved our ability to detect true biological variance was simple scaling of read counts by sequencing depth, which does not operate under of the assumptions inherent to the aforementioned methods^16^. For most studies, adjusting for sequencing by generating RPM values as we did here, or by generating transcripts per million (TPM) values by additionally adjusting for transcript length, should be adequate to remove technical variance while leaving the ability to detect true biological variance intact, especially in analyses with modest to large sample sizes^29^, which is becoming increasingly common in transcriptomic biomarker investigations due to the decreasing cost of bulk sequencing.

It could be argued that a limitation to this study is that we only experimentally assessed the effects of the tested normalization strategies on our ability to detect biological variance in terms of expected differences in whole blood mRNA levels driven by underlying differences in cellular composition, and not differences in whole blood mRNA levels driven by cell-level differences in nuclear transcription. To directly assess the latter, we would have needed a priori knowledge of true transcriptional differences between specimens, or ideally, to have used a spike-in strategy. However, the absolute mRNA level for any given gene in a bulk sample is collectively determined by the relative proportions of functionally distinct cell populations present in the sample, and the nuclear transcriptional state of said gene within the cell populations themselves^28^; given that tissue-level transcript abundance is cumulatively dependent on both factors, if the variance contributed by cell type admixture effects is not accurately quantified in an experiment, then further variance stemming from true transcriptional differences will also be harder to reliably detect. Thus, because MRN, TTM, and quantile normalization dramatically disrupted the baseline global distributions of transcript abundance which were biologically expected based on sample leukocyte composition, we can assume that their use for normalization would also interfere with the ability to detect differences in whole blood mRNA levels driven by underlying cell-level differences in rates of nuclear transcription, not just those driven by cell-type admixture effects.

One obvious caveat to the conclusions we have drawn here lies in that we evaluated the performance of the tested normalization strategies within the context of investigations focused on discovery of RNA-based biomarker panels, where the goal is typically to identify diagnostically targetable absolute differences in mRNA levels that are directly observable at the level of whole blood, as opposed to making inferences about the underlying nuclear transcriptional state of the composite leukocyte pool, which may be the case in studies more focused purely on evaluating physiologic or pathophysiologic mechanisms. Two of the normalization strategies we tested, MRN and TMM normalization, were actually conceptualized with the latter application in mind; they were designed with the intention of helping reveal true nuclear transcriptional state by controlling for potential sequencing-specific biases associated with the influence of the read counts of most highly expressed genes on the read counts of the remainder of the genome given the finite number of total reads that are generated from a sample^30, 31^. While such correction may be more warranted in a physiologic experiment aimed at understanding nuclear transcriptional state, given the degree to which the core assumptions of these normalization methods were violated in our analyses, we would still have reservation about their use with whole blood data for any experimental aim. While these methods have exhibited strong performance in prior benchmarking studies which have utilized simulated data or real data generated from various human and non-human solid tissues^11, 32–34^, these studies did not evaluate them for specific use with data generated from whole blood as we did here.

While our findings have direct implications for informing data normalization strategies in biomarker-centric whole blood gene expression analyses, they also shed light on other caveats associated with the analysis of whole blood gene expression data in general. For example, the results of the permutation analysis we performed when assessing gene-level expression differences between specimens from donors with differing leukocyte counts suggest that neutrophil-to-lymphocyte ratio is likely by a wide margin the single largest determinant of the overall pattern of gene expression observed at the level of whole blood. While this result is not necessarily surprising, and aligns well with prior reports by our group and others^35–39^, continuing to experimentally illustrate and emphasize this phenomenon as we have done here is important, as an alarming proportion of the vast number of papers currently being published reporting the results of experiments performed with whole blood gene expression data fail to account for potential inter-specimen differences in leukocyte composition in their experimental designs, or to discuss the influence of any such differences on the results; many of these papers interpret the results of their analyses under the flawed assumption that a majority of the observed gene expression differences are driven by true changes in nuclear transcription, ignoring the fact that many are likely a result of underlying changes in specimen cellularity, which is undoubtedly leading to widespread biological misinterpretation of findings. Furthermore, an additional insight our results provide outside of the primary aim of our analyses is that many of the commonly employed data normalization techniques which we evaluated should likely be avoided in studies of whole blood gene expression data using cellular deconvolution methods, as their use could clearly interfere with the accuracy of cell type enumeration.

Our collective findings highlight the pitfalls associated with the application of widely-used RNA sequencing data normalization strategies which rely on the assumption of homogeneous transcriptome composition across specimens in biomarker discovery studies performed with whole blood. Given our results, such normalization techniques should be avoided in future investigations in lieu of others that are not confounded by inter-specimen differences in transcriptome composition associated with heterogeneity in donor leukocyte counts.

## Materials and methods

### Analysis of gene expression data originating from isolated neutrophils and lymphocytes

RNA sequencing data generated from isolated human neutrophils, naive CD4+ T cells, naive CD8+ T cells, memory CD4+ T cells, memory CD8+ T cells, naive B cells, and memory B cells originally sampled from the peripheral blood of healthy donors by Uhlen et al.^40^ were retrieved as gene-level summarized TPM values from the Human Protein Atlas (https://www.proteinatlas.org/about/download). Median TPM values observed in neutrophil samples and the total pool of lymphocyte samples were used directly for fold difference calculations.

Microarray data originating from isolated human neutrophils, TH1 CD4+ T cells, TH2 CD4+ T cells, memory CD4+ T cells, and B cells originally sampled from the peripheral blood of healthy donors by Mackay et al.^41^ were retrieved as raw intensity values with corresponding present-absent calls from the National Center for Biotechnology Information (NCBI) Gene Expression Omnibus (GEO) via accession number GSE3982. Median raw intensity values observed in neutrophil samples and the total pool of lymphocyte samples were used directly for fold difference calculations. Note that raw values were used in this analysis because the most commonly used microarray data-normalization approaches, such as quantile normalization, would have likely masked the true biological structure of the data if applied to samples with dramatically different transcriptome composition, in a similar manner as the RNA sequencing data normalization methods we evaluated.

### Patients and blood sampling

Patients were recruited in the emergency department at Dell-Seton Medical Center (Austin, Tx) as part of a larger acute care biomarker discovery investigation, and presented with a broad range of medical conditions. Venus whole blood was collected via both PAXgene and K_2_EDTA vacutainers, prior to initiation of any treatment. PAXgene vacutainers were moved to − 80 °C for storage until downstream RNA isolation, while K_2_EDTA vacutainers were used immediately for hematology analysis. Donor clinical and demographic information was retrospectively retrieved from the electronic medical record. All methods were carried out in accordance with relevant guidelines and regulations, and were approved by the Institutional Review Board of Dell-Seton Medical Center. Written informed consent was obtained from all subjects or their authorized representatives prior to any study procedures.

### White blood cell differential

White blood cell differential was immediately assessed in EDTA-treated blood via four angle optical flow cytometry on the Cell-Dyn Sapphire automated clinical hematology system using the Cell-Dyn WBC reagent pack (Abbott Diagnostics, Santa Clara, CA). Relative counts of leukocyte subpopulations were generated by dividing absolute subpopulation cell counts by the absolute total leukocyte count. The neutrophil-to-lymphocyte ratio was calculated as relative neutrophil count divided by relative lymphocyte count.

### RNA sequencing of whole blood

Total RNA was isolated from archived PAXgene-stabilized whole blood using the PAXgene IVD Blood RNA Kit (PreAnalytiX GmbH). RNA purity was assessed using spectrophotometry (NanoDrop 3300, Thermo Scientific), RNA concentration was assessed using fluorometry (Qubit RNA broad range assay kit, Thermo Scientific), and RNA integrity was assessed using chip capillary electrophoresis (2100 Bioanalyzer, Agilent Technologies, Inc).

Ribosomal RNA and globin mRNA-depleted cDNA libraries were prepared from 500 ng of total RNA using the illumina TruSeq Stranded Total RNA Ribo-Zero Globin kit (Illumina, Santa Clara, CA). Paired-end 150 bp sequencing was performed on the illumina NovaSeq 6000 platform. Pre-processing of raw data was performed using R (R Project for Statistical Computing)^15^. Low quality reads were filtered, and remaining reads were aligned to human reference genome GRCh38 using the HISAT2 pipeline via the ‘rhisat2’ package^42^. Counts of mapped reads were summarized at the gene level using the featureCounts() function of the ‘Rsubread’ package^43^.

### RNA sequencing data normalization

All data normalization was carried out using R. Scaling of raw read counts to produce RPM values was performed using basic scripts. MRN normalization was performed via the ‘DEseq2’ package^13^, using a combination of the DESeq() and counts() functions under default settings to respectively generate scaling factors and retrieve scaled read counts. TMM normalization was performed via the ‘edgeR’ package^14^, using a combination of the calcNormFactors() and cpm() functions under default settings to respectively generate scaling factors and retrieve scaled read counts. Quantile normalization was performed via the ‘preProcess’ package using normalize.quantiles() function with ties in read counts being broken at random.

### Cellular deconvolution of whole blood gene expression profiles

RNA sequencing data generated from isolated human neutrophils, naive CD4+ T cells, naive CD8+ T cells, memory CD4+ T cells, memory CD8+ T cells, naive B cells, memory B cells, monocytes, natural killer cells, and eosinophils originally sampled from the peripheral blood of healthy donors by Uhlen et al.^40^ were retrieved as gene-level summarized TPM values from the Human Protein Atlas (https://www.proteinatlas.org/about/download) and used to select the WBC4.126 marker gene list used for deconvolution. Final marker genes for neutrophils, total lymphocytes, monocytes, and eosinophils were selected by filtering the total pool of detected genes to retain those which exhibited at least 100-fold higher expression levels in one target cell population relative to the others, and also a median expression level of 1.0 TPM within the target cell population of maximal expression. In said analysis, inter-cell population fold differences were calculated from weighted median TPM values, which were used to avoid bias associated with differing numbers of samples associated with each target cell population.

For deconvolution, principal components analysis was used to produce an inferred count for each target cell population based on the whole blood read counts of its associated marker genes using the collapserows() function of the ‘WGCNA’ package^44^ for R as described by our group previously^45^, with cell count values based solely on the elgenvector capturing the most variance. The resultant inferred counts of each cell population were arbitrarily scaled from zero to one using unity-based normalization.

### Statistical analyses

All statistics were carried out using the R ‘stats’ package. Mann–Whitney U-test or Kruskal–Wallis rank test was used for the comparison of continuous variables where appropriate. Spearman’s rho was used to assess the strength and significance of correlational relationships. Permutation analyses were carried out using basic scripts and the sample() function of base R. Bootstrap statistical comparisons were carried out using the percentile bootstrap method^46^, and were similarly implemented using basic scripts and the sample() function of base R. Sample sizes were arbitrarily determined. In the case of all statistical testing, the null hypothesis was rejected when p < 0.05. The parameters of all statistical tests performed are outlined in detail within the figure legends.

### R versioning

All R analyses were carried out using R version 4.3.

### Supplementary Information

Supplementary Information.

## Data Availability

Data are available from the National Center for Biotechnology Information (NCBI) via BioProject accession number PRJNA949611. Raw sequencing data are available as .fastq files via individually linked Sequence Read Archive (SRA) records, and can be downloaded in bulk via the SRA run selector. Demographic information, as well as neutrophil-to-lymphocyte ratios, for all 138 donors are available via the attributes slots of linked individual BioSample records, and can be downloaded along with sequencing data as metadata using the SRA run selector.
